# Planar cell polarity genes Frizzled3a, Vangl2, and Scribble are required for spinal commissural axon guidance

**DOI:** 10.1186/s12868-016-0318-z

**Published:** 2016-12-12

**Authors:** Simon D. Sun, Ashley M. Purdy, Gregory S. Walsh

**Affiliations:** Department of Biology, Virginia Commonwealth University, 1000 West Cary Street, Richmond, VA 23284 USA

**Keywords:** Fzd3a, Scrib, PCP, Commissural pathfinding, Midline

## Abstract

**Background:**

A fundamental feature of early nervous system development is the guidance of axonal projections to their targets in order to assemble neural circuits that control behavior. Spinal commissural neurons are an attractive model to investigate the multiple guidance cues that control growth cone navigation both pre- and post-midline crossing, as well as along both the dorsal–ventral (D–V) and anterior–posterior (A–P) axes. Accumulating evidence suggests that guidance of spinal commissural axons along the A–P axis is dependent on components of the planar cell polarity (PCP) signaling pathway. In the zebrafish, the earliest born spinal commissural neuron to navigate the midline and turn rostrally is termed commissural primary ascending (CoPA). Unlike mammalian systems, CoPA axons cross the midline as a single axon and allow an analysis of the role of PCP components in anterior pathfinding in single pioneering axons.

**Results:**

Here, we establish CoPA cells in the zebrafish spinal cord as a model system for investigating the molecular function of planar cell polarity signaling in axon guidance. Using mutant analysis, we show that the functions of Fzd3a and Vangl2 in the anterior turning of commissural axons are evolutionarily conserved in teleosts. We extend our findings to reveal a role for the PCP gene *scribble* in the anterior guidance of CoPA axons. Analysis of single CoPA axons reveals that these commissural axons become responsive to PCP-dependent anterior guidance cues even prior to midline crossing. When midline crossing is prevented by *dcc* gene knockdown, ipsilateral CoPA axons still extend axons anteriorly in response to A–P guidance cues. We show that this ipsilateral anterior pathfinding that occurs in the absence of midline crossing is dependent on PCP signaling.

**Conclusion:**

Our results demonstrate that anterior guidance decisions by CoPA axons are dependent on the function of planar cell polarity genes both prior to and after midline crossing.

## Background

A fundamental feature of neural circuit assembly is the complex guidance of axonal processes to their target. Extensive work in both vertebrate and invertebrates have revealed evolutionarily conserved molecular guidance cues that control the trajectory of growing axons along both the dorsal–ventral (D–V) and the anterior–posterior (A–P) axes [1, 2]. Owing to their navigation along both axes in the developing nervous system, commissural neurons in the spinal cord represent a well-studied model system for how neurons respond to multiple guidance cues [3, 4].

In the vertebrate spinal cord, dorsally-located commissural neurons are first attracted to the ventral midline through the action of floor-plate-derived chemoattractants, namely Netrin and Sonic Hedgehog (SHH) [5–9]. Upon entry into the midline, they lose responsiveness to attractive Netrin-DCC signals and acquire sensitivity to the midline-produced repellants, such as Slits and Semaphorins [10–13]. In response to Slit-Robo signaling, commissural axons are expelled from the midline [14, 15]. Lastly, responsiveness to anterior–posterior guidance cues, including the morphogens Wnt and SHH, specify rostral or caudal turning along the longitudinal axis [4].

The responsiveness of spinal commissural axons to Wnt ligands is mediated by activation of the non-canonical/planar cell polarity (PCP) signaling pathway. As first defined in *Drosophila*, PCP coordinates the uniform orientation of cells within the plane of the epithelium [16, 17]. The network of PCP proteins described for vertebrates include the core members: Frizzled (Fzd), the seven-pass transmembrane domain protein, Celsr (cadherin EGF LAG seven-pass G-type receptor), an atypical cadherin with seven-pass transmembrane domains, the four-pass transmembrane protein Van Gogh-like (Vangl), and the cytoplasmic proteins Dishevelled (Dsh), and Prickle (Pk). Downstream of the core PCP proteins in both fly and vertebrates is Scribble (Scrib), a member of the leucine-rich repeat and PDZ (LAP) family of proteins. Scrib has now been shown to be required for a broad range of processes regulated by PCP, including convergence-extension (CE) cell movements, neural tube closure, orientation of inner-ear mechanosensory hair cells, and neuronal migration [18–24], however, an involvement in commissural axon guidance has not been reported.

A role for Wnt-Frizzled signaling in the guidance of commissural axons is supported by the observation that exogenously applied Wnt ligands attract commissural axons in spinal cord explants [25, 26]. Dorsal spinal commissural axons were found to lose A–P directionality and turn randomly, either anteriorly or posteriorly, after midline crossing in *Fzd3* mutant mice and in chick following after *Fzd3* knockdown [25, 27]. The observation in mouse that Wnt4 is expressed in a high-anterior to low-posterior gradient is consistent with the notion that Wnts may act as a diffusible chemo-attractant guiding commissural growth cones to turn anteriorly after midline crossing [25]. In chick, although Wnt ligands are not expressed in a gradient, SHH was shown to be present in a decreasing posterior-to-anterior gradient that mediates the graded expression of the Wnt-antagonist, Secreted frizzled-related protein (SFRP)1, that sculpts a decreasing Wnt activity gradient from anterior to posterior [26]. Recently, several additional PCP components, *Vangl2*, and *Celsr3,* were shown to be necessary for the anterior turning of post-crossing axons in mice [28], and chick [29], indicating that Wnt-PCP signaling controls the anterior steering of commissural axon growth cones.

Here, we have examined the requirement for PCP components in the anterior guidance of a single commissural axon in the spinal cord of zebrafish. Several populations of commissural neurons are born in every segment of the zebrafish spinal cord [30, 31]. These can be distinguished based on morphology and cell body position within the spinal cord. The commissural primary ascending (CoPA) neuron, found at an average of one per hemisegment, has a dorsally located cell body with a single axon that crosses the midline and projects anteriorly to target cells in the contralateral hindbrain [30–32]. The CoPA neuron is the first commissural neuron to be born and send its axon across the midline and functions as the pioneer commissural axon in the zebrafish spinal cord [32].

In this study, we show that the function of the PCP pathway in anterior guidance of commissural axons is evolutionarily conserved across vertebrates. We have determined that in addition to *fzd3a* and *vangl2, scrib* is required for the proper anterior–posterior guidance of individual pioneer commissural axons. Unlike mammalian systems, in which commissural axons become responsive to anterior guidance cues only after crossing the midline, we show that PCP components influence anterior guidance of CoPA commissural axons as they extend both pre- and post-midline crossing. When midline crossing is eliminated, CoPA axons can still respond to anterior–posterior guidance cues and extend appropriately in a rostral direction within the ipsilateral spinal cord. We show that this ipsilateral anterior guidance, that occurs when midline crossing is prevented, is dependent on the function of PCP components.

## Methods

### Fish strains and mutants

Zebrafish (*Danio rerio*) were maintained according to standard procedures and were staged as previously described [33]. The *fzd3a* mutant was originally described as *off*-*limits*/*olt* ^*rw689*^ [34]. The *vangl2*/*trilobite* mutant was originally described as *tri* ^*m209*^ [35]. The *scrib* mutant was originally described as *landlocked*/*llk* ^*rw468*^ [19]. The *pk1b* mutant was originally described as *pk1b* ^*fh122*^ [36].

### Morpholino Injections

3 ng of *dcc* translation-blocking morpholino (GATATCTCCAGTGACGCAGCCCAT; start codon complement underlined) was injected at the one-cell stage using an ASI MPPI-3 (Applied Scientific Instrumentation) pressure injector. Morpholinos were obtained from Gene Tools, LLC.

### Immunofluorescence

The 3A10 antibody from DSHB was used at a concentration of 1:10 on embryos at 31 hpf that had been fixed in 4% paraformaldehyde (in 1× PBS) overnight at 4 °C. Embryos were washed in PBST (1× PBS with 0.25% Triton X-100), permeabilized with ice-cold acetone, and washed again with PBST. Embryos were blocked with PBST + 10% Goat Serum + 4% BSA at room temperature for 1 h. Alexa Fluor 568 Goat Anti-Mouse IgG (H + L) secondary antibody (catalog number A11031, Life Technologies) was added at a concentration of 1:200 overnight at 4 °C. Embryos were washed 5 × 30 min in PBST (1× PBS with 0.25% Triton X-100) in between antibody incubations. The embryos were then sequentially dehydrated in 25, 50, and 75% glycerol in 1× PBS.

### Microscopy

After immunofluorescence, the yolks of the embryos were removed by micro-dissection. Embryos were mounted on coverslips on their sides for lateral visualization of the spinal cords. Embryos were mounted in 100% glycerol.

Confocal images of labeled CoPA neurons were obtained on a Carl Zeiss Spinning Disk Laser Confocal Observer Z1. To obtain images of CoPA contralateral axon pathfinding, confocal projections were made from optical sections obtained by imaging from one side of the spinal cord through to the other side of the spinal cord. To visualize midline crossing by CoPA axons, orthogonal projections were made of the same confocal image stacks.

### Quantification of anterior–posterior guidance of CoPA axons

CoPA neurons were scored for the anterior or posterior direction of post-midline crossing axons. Only CoPA axons caudal to the 9th somite were scored for reproducibility. Analysis of pre-crossing axons was achieved by drawing a line perpendicular to the A–P axis at the axon hillock of each CoPA cell. A second line was drawn from the axon hillock to the point of entry at the floorplate. If the angle of the two lines was greater than 3°, the axon fiber was considered to have an anterior or posterior direction bias. Pearson’s Chi square test was utilized to test percentages for statistical significance. We measured the distance travelled by CoPA axons within the floorplate along the A–P axis from lateral view confocal stacks using image analysis software (Zeiss). The commissure can be defined as the area of the midline occupied by CoPA axons along the longitudinal axis, which is devoid of ventrally-projecting ipsilateral axon, or the dorsally projecting contralateral axon. Statistical significance for commissure length was determined using a two-tailed Student’s *t* test. All statistical tests were conducted with JMP11 statistical software provided by Virginia Commonwealth University.

## Results

### Planar cell polarity proteins are required for anterior guidance of CoPA axons

**Fig. 1:**
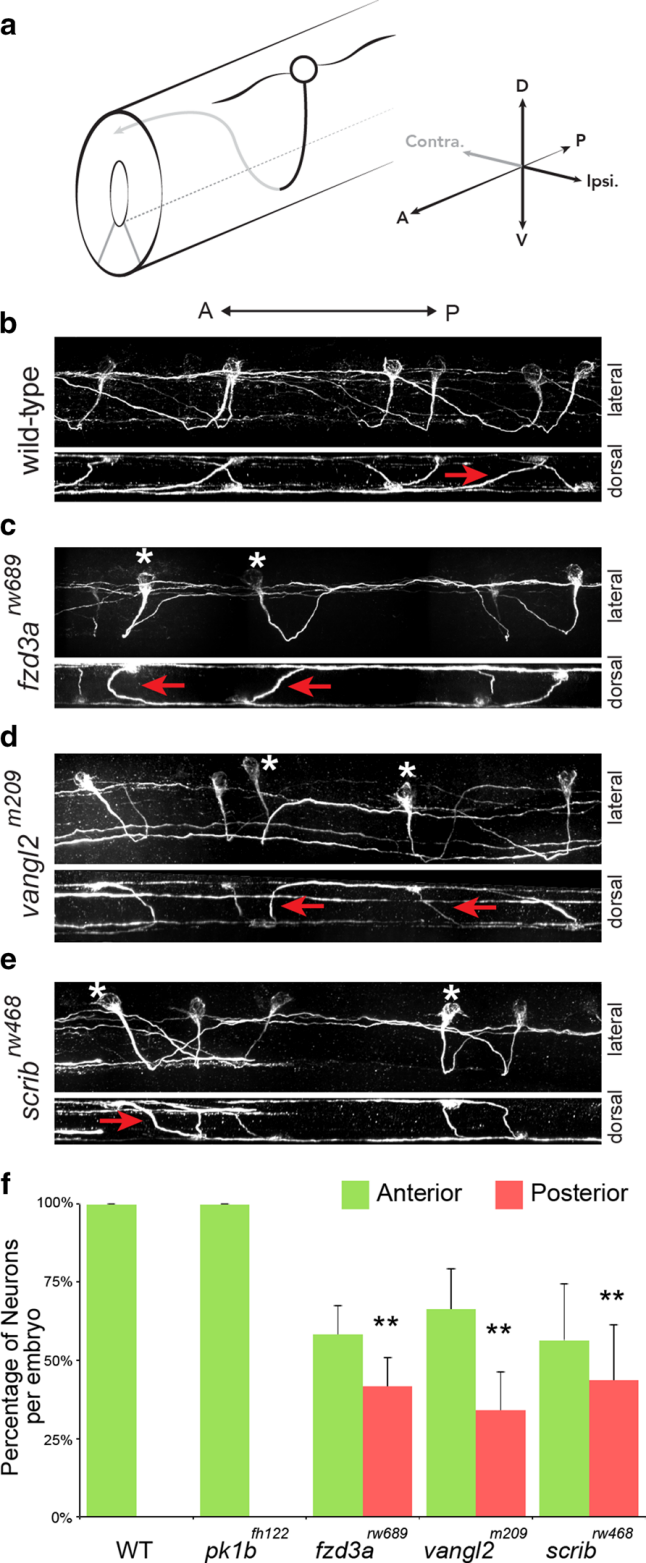
PCP genes are required for anterior pathfinding of CoPA axons after midline crossing. **a** Illustration of a CoPA neuron in the spinal cord. *Black line* represents ipsilateral ventral projection, *Gray line* indicates dorso-anterior trajectory after midline crossing. CoPA neurons have two major dendrites, one projecting anteriorly, and the other projecting posteriorly, **b** confocal micrographs of 3A10 immunofluorescence shows the pathfinding of CoPA axons in multiple segments of the spinal cord in wild-type (WT) embryos. In the lateral view (*upper panel*), all CoPA axons project dorso-anteriorly after midline crossing. Anterior is to the *left*, dorsal is *up*. In the *lower panel*, a dorsal view of the same spinal cord shows the midline crossing trajectory of CoPA axons (*red arrows*), **c**–**e** in *fzd3* ^*rw689*^, *vangl2* ^*m209*^, and *scrib* ^*rw468*^ mutant embryos, approximately half of CoPA axons fail to turn anteriorly after crossing the midline. *Asterisks* mark affected CoPA cells that turn posteriorly, inappropriately. Lateral views of the same spinal cords show that affected CoPA axons still cross the midline (*red arrows*), **g** quantification of anterior–posterior trajectories of CoPA axon after midline crossing per embryo. Error indicates SD. **p < 0.01 versus WT

We examined CoPAs from somite levels 9–17 at 31 hpf, a timepoint in which CoPA pathfinding is largely complete. CoPA neurons were visualized using immunostaining with the 3A10 antibody. At this developmental timepoint, the only neuronal cell bodies to be labeled by 3A10-immunostaining in the spinal cord are CoPA cells, but also labels the axons of descending Mauthner neurons in the medial longitudinal fascicle (MLF) [15, 37, 38]. In wild-type embryos, we found that CoPA cells were located in the dorsal region of the spinal cord with approximately 0–2 cell bodies per segment (Fig. 1a, b). These neurons have a single unbranching axon that projects ventrally and crosses the midline in the floor of the spinal cord. After midline crossing, CoPA axons extend simultaneously dorsally away from the midline and anteriorly towards the head (Fig. 1a, b). In this last stage, CoPA axons grow in a dorso-anterior direction, ascending at an oblique angle to the dorsal spinal cord where it joins other CoPA axons from more caudal segments in the dorsal longitudinal fasciculus (DLF). These observations are consistent with previous reports detailing CoPA cell morphology [15, 30, 32].

To determine whether core components of the PCP signaling pathway are essential in the pathfinding of CoPA axons, we first examined the trajectory of CoPA commissural axons in zygotic *fzd3a* ^*rw689*^ mutants that have a missense mutation in the extracellular cysteine-rich domain (CRD) that affects the membrane association of Fzd3a [34]. Expression of *fzd3a* is ubiquitous in the developing nervous system [34, 39]. *fzd3a* ^*rw689*^ mutants are morphologically normal but exhibit PCP-related defects in the nervous system including a defect in facial branchiomotor neuron migration in hindbrain [34]. In *fzd3a* ^*rw689*^ mutants, CoPA neurons are specified correctly, but display a defect in anterior–posterior guidance. Although many CoPAs behave similar to that described for wild-type embryos, almost half of CoPA axons in *fzd3a* ^*rw689*^ mutants turn posteriorly, inappropriately (42 ± 9%; 7 embryos; *n* = 79 neurons) (Fig. 1c). This randomized A–P turning phenotype was observed in all *fzd3a* ^*rw689*^ mutants making the phenotype fully penetrant. Despite a failure in A–P directionality, CoPA axons in *fzd3a* ^*rw689*^ mutants undergo normal ventral extension and can be observed to cross the midline normally as seen in dorsal projections of confocal images (Fig. 1c). Regardless of anterior or posterior turning, CoPA axons in *fzd3a* ^*rw689*^ mutants pathfind dorsally to join other CoPA axons traveling in the DLF (Fig. 1c). This finding demonstrates that Fzd3a is not required for dorsal–ventral guidance decisions of CoPA axons before or after midline crossing. Since we can trace the entire axon of single CoPA cells in *fzd3a* ^*rw689*^ mutants, we observed that axons of affected CoPAs do not stall or wander, but extend for long distances (albeit in the wrong direction). Taken together, our data show that Fzd3a is not required for axon growth per se, but is required for proper guidance decisions of CoPA axons along the anterior–posterior axis.

To further test whether PCP components are responsible for proper A–P guidance of commissural axons, we analyzed zygotic *vangl2* ^*m209*^ mutants. Similar to *fzd3a*, *vangl2* is expressed ubiquitously in the developing nervous system [35, 40, 41]. *trilobite*/*vangl2* mutants display a shortened and wider body axis due to defective convergence and extension (CE) cell movements [35, 42, 43]. Although maternal-zygotic *trilobite*/*vangl2* mutants have neural tube morphogenesis defects, neural tube formation is largely normal in zygotic *trilobite*/*vangl2* mutants but do have a widened floorplate due to convergence defects [44, 45]. Despite this, CoPA cells are born at the proper frequency along the length of the spinal cord in *vangl2* ^*m209*^ mutants. CoPA cells also exhibit a defect in anterior turning in *vangl2* ^*m209*^ mutants with 34 ± 13% (15 embryos; *n* = 94 neurons) of CoPA axons extending posteriorly inappropriately in each embryo (Fig. 1d). Regardless of anterior or posterior trajectory, CoPA axons in *vangl2* ^*m209*^ mutants cross the midline appropriately and pathfind dorsally after midline crossing, consistent with the notion that loss of planar cell polarity does not affect dorsal–ventral pathfinding of commissural axons (Fig. 1d).

We also examined CoPA pathfinding in *prickle1b* (*pk1b* ^*fh122*^) mutants, that have a missense mutation in the farnesylation motif (CAAX domain) that abrogates its function [36], but found no significant difference in CoPA pathfinding compared to wildtype embryos (7 embryos; *n* = 76 neurons) (Fig. 1f). This is unsurprising, since *pk1b* shows little to no expression in the spinal cord [36, 46].

Finally, we examined CoPA guidance in zygotic *scrib* ^*rw468*^ mutants [19]. Similar to *fzd3a, scrib* is expressed ubiquitously in the developing nervous system [19]. The *scrib* ^*rw468*^ allele contains a point mutation that results in a pre-mature stop codon in the leucine rich repeat (LRR) domain of Scrib [19]. Unlike *trilobite*/*vangl2* mutants, zygotic *scrib* ^*rw468*^ mutants are morphologically normal and do not show any defects in CE movements [19]. We found that CoPA axons in zygotic s*crib* ^*rw468*^ mutants also showed defects in anterior–posterior guidance with approximately half of these turning posteriorly, inappropriately (44% ± 18; 14 embryos; *n* = 104 neurons) (Fig. 1e). Similar to *fzd3a* ^*rw689*^ mutants, affected CoPA axons in s*crib* ^*rw468*^ mutants executed normal D–V guidance and extended for long distances along the A–P axis, irrespective of the direction that they were travelling (Fig. 1e). This is the first demonstration to our knowledge that Scrib is required for the A–P guidance of spinal commissural axons in vertebrates.

Taken together, our findings demonstrate that the PCP components *fzd3a*, *vangl2*, and *scrib* are all required for the anterior guidance of the CoPA commissural axons. Given that CoPA axons grow long distances, both anteriorly or posteriorly, in PCP mutants, our findings also suggest the presence of other guidance molecules that control CoPA axon trajectories along the anterior–posterior axis.

### Commissure length is normal in PCP mutants

**Fig. 2:**
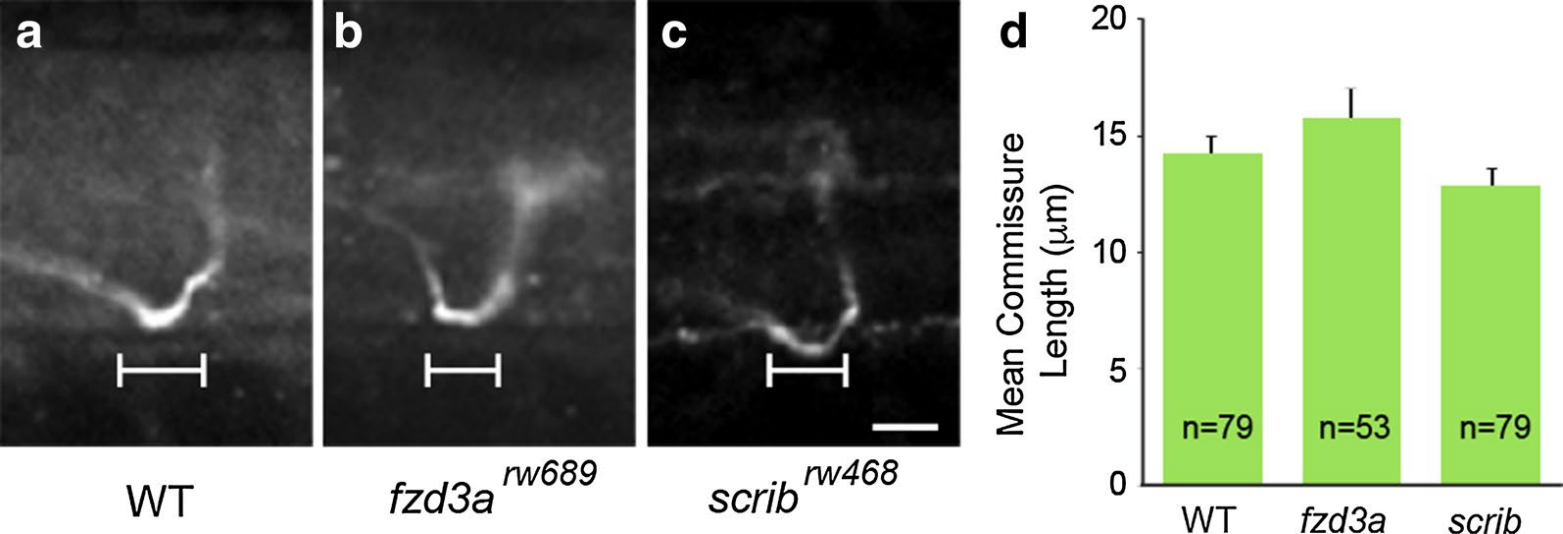
Commissure formation is not affected by loss of *fzd3a* or *scrib*. **a**–**c** Confocal micrographs showing lateral view of commissures from individual CoPA axons in WT, *fzd3a* ^*rw689*^, and *scrib* ^*rw468*^ embryos. Anterior is to the *left*, dorsal is *up*, **d** Measurements of distance traveled by axon within the midline of the spinal cord in the anterior–posterior axis. No significant difference was found in the length of commissures in WT, *fzd3a* ^*rw689*^, and *scrib* ^*rw468*^ embryos. *n* number of neurons counted. *A* two-tailed Student’s t test was used for statistical analysis. Error indicates SEM

To ensure that loss of PCP components has no effect on dorsal–ventral guidance, we evaluated commissural architecture in *fzd3a* ^*rw689*^ and s*crib* ^*rw468*^ mutants. Recently, it was reported that Slit-Robo signaling was required for proper midline exit of CoPA axons [15]. In *robo2* and *robo3* mutants, CoPA axons remain in the midline, travelling anteriorly, for significantly longer distances compared to wild-type embryos [15]. We quantified the distance travelled by CoPA axons within the floorplate along the longitudinal axis from lateral views of the spinal cord. Since *trilobite*/*vangl2* mutants have CE defects, we confined the rest of our analysis to *fzd3a* and *scrib* mutants. We found no significant difference in the extent of growth within the floorplate in *fzd3a* ^*rw689*^ or s*crib* ^*rw468*^ mutants when compared with wild-type embryos (Fig. 2a–d). Thus, CoPA axons in PCP mutants do not spend more time travelling within the floorplate than wild-type CoPAs, supporting the notion that dorsal–ventral pathfinding, mediated primarily by Netrin-Dcc and Slit-Robo signaling, is unaffected by the loss of the PCP signaling pathway.

### PCP proteins influence CoPA axon pathfinding before midline crossing

**Fig. 3:**
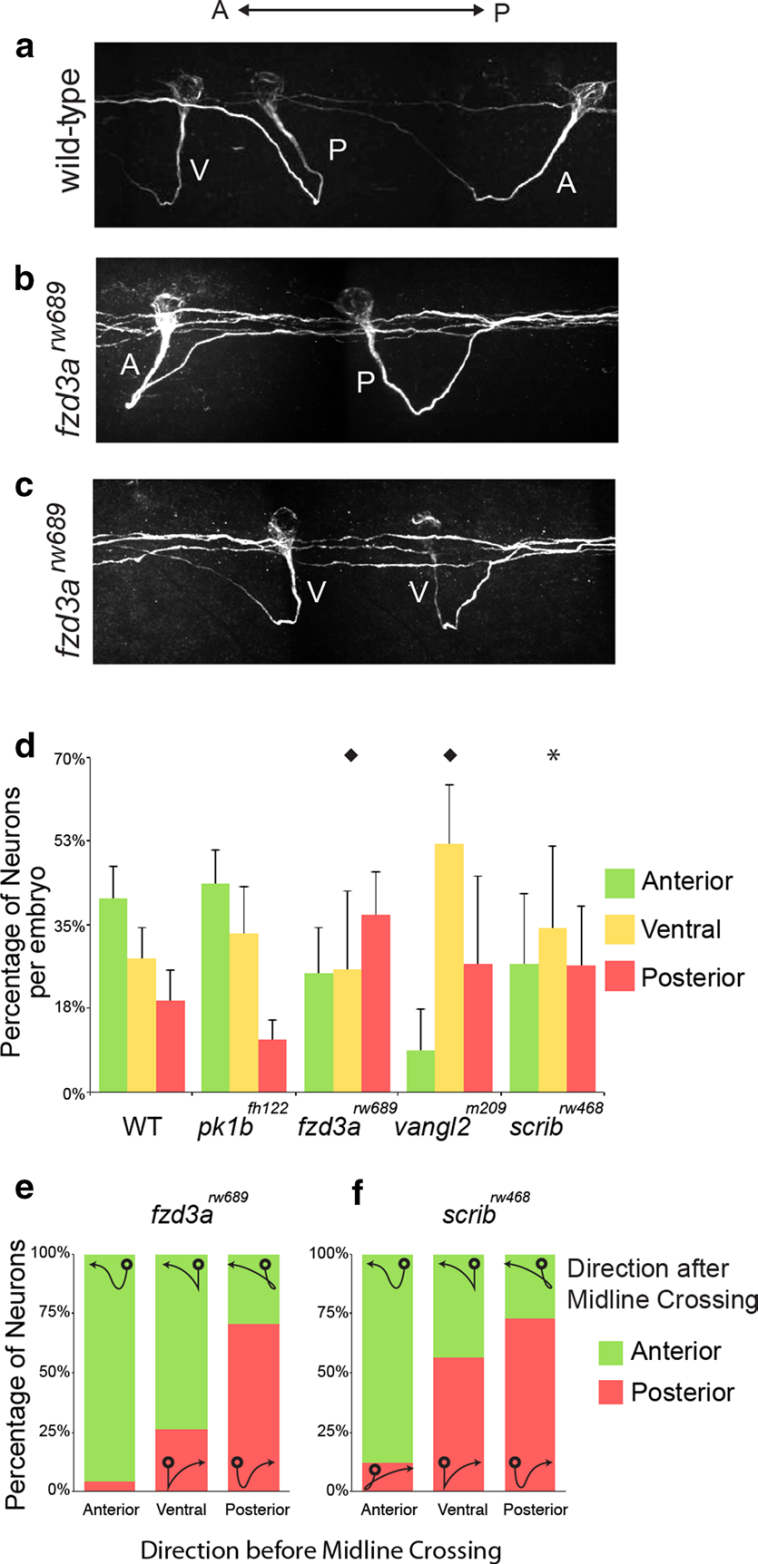
Pre-crossing commissural axon pathfinding is influenced by PCP components. **a**–**c** Confocal micrographs of CoPA axon pathfinding in WT and *fzd3a* ^*rw689*^ embryos illustrating examples of CoPA axon trajectories. Anterior is to the *left*, dorsal is *up*. **a** CoPA axons have an anterior–posterior bias as they extend ventrally towards the midline. The majority of CoPA axons traverse ventro-anteriorly (A), straight ventrally (V), while a small proportion extend ventro-posteriorly (P). All CoPAs turn anteriorly by the time they have reached the contralateral spinal cord in wild-type embryos, **b** in *fzd3a* ^*rw689*^ mutants, examples of pre-crossing CoPA axons that project ventro-posteriorly (P) and continue posteriorly after midline crossing, and a small number that project ventro-anteriorly before turning posteriorly after midline crossing, **c** in *fzd3a* ^*rw689*^ mutants, pre-crossing CoPA axons that project straight ventrally (V) are more likely to make random A–P guidance as post-crossing fibers, **d** quantitation of the midline crossing points relative to cell body position among genotypes per embryo (*p < 0.05; ♦p < 0.01; Pearson Chi square test). **e**, **f** distribution of post-crossing axon trajectory relative to the pre-crossing directional bias in *fzd3a* ^*rw689*^ and *scrib* ^*rw468*^ mutants. Superimposed on the graph are representations of the appearance of CoPA trajectories

CoPA neurons have axons that project ventrally before midline crossing. Previous studies using retrograde tracers reported that these axons project either straight ventrally or at an oblique, anteriorly-directed angle, even prior to crossing the midline [31]. We quantified this and found, indeed, that the majority of pre-crossing axons (78%, *n* = 107; 7 embryos) extend either straight ventrally from the cell soma or have a ventro-anterior trajectory as they extend towards the midline (Fig. 3a, d). Interestingly, we did observe a small proportion of pre-crossing fibers (22%) that initially project in a ventro-posterior direction and cross the midline at a position that is slightly caudal to the cell soma (Fig. 3a, d). Irrespective of the initial directional bias of pre-crossing axons, we observed that all CoPA axons had turned anteriorly by the time they had crossed the midline in wild-type embryos (Figs. 1b, 3a). In fact, some CoPA axons that initially project ventro-posteriorly can be found to correct course (turn anteriorly) even prior to reaching the midline. Taken together, our observations indicate that CoPA axons appear to become responsive to anterior–posterior guidance cues prior to midline crossing.

We then analyzed whether PCP proteins play a role in A–P guidance of pre-crossing CoPA axons. We found a statistically significant difference in the midline-crossing position of CoPA axons in *fzd3a* ^*rw689*^ mutants (*n* = 84, 7 embryos*; p* < 0.01, Pearson’s Chi square test)*, vangl2* ^*m209*^ mutants (*n* = 40, 5 embryos; *p* < 0.01), and *scrib* ^*rw468*^ mutants (*n* = 145, 15 embryos*; p* < 0.05), with an increase in the proportion of axons that crossed the midline in a position that was vertical or posterior to the cell soma compared to wild-type embryos (Fig. 3b–d). As expected, quantitation of the midline crossing point in *pk1b* ^*fh122*^ mutants (*n* = 93; 7 embryos) revealed no significant difference compared to wild-type embryos. Therefore, components of the PCP signaling pathway contribute to the anterior guidance of CoPA axons even before they cross the midline.

We next sought to determine if there was a relationship between the pre-crossing axon trajectory and the post-crossing guidance decision of CoPA axons in PCP mutant embryos. Since CoPA axons in wild-type embryos always make the correct anterior guidance decision by the time they have crossed the midline, we focused our analysis on CoPA axon trajectories in *fzd3a* ^*rw689*^ and *scrib* ^*rw468*^ mutants. We found that if pre-crossing fibers have an anterior component to their growth then they most-often continue to grow anteriorly after crossing the midline (*fzd3a*: 95%; *n* = 22 cells; *scrib*: 88%; *n* = 50 cells) (Fig. 3b, c, e). On the other hand, if PCP-deficient CoPA axons had a posterior bias as pre-crossing fibers, they were more likely to continue projecting posteriorly after midline crossing (*fzd3a*: 70%; n = 34 cells; *scrib*: 72%; n = 44 cells) (Fig. 3e). Those CoPA axons that cross the midline straight ventral to the cell soma, however, tend to make more randomized guidance choices after crossing the midline in *fzd3a* ^*rw689*^ and *scrib* ^*rw468*^ mutants (Fig. 3c, e). These observations suggest that PCP signaling may influence the initial orientation of axon outgrowth from the cell soma favoring an anterior bias. In the absence of PCP signaling components, the initial bias of CoPA axon outgrowth is more randomized and CoPA axons tend to maintain their initial bias in that direction as they continue to grow and cross the midline. Taken together, these data suggest that PCP signaling influences the anterior–posterior guidance of CoPA axons both pre- and post-midline crossing.

### Anterior guidance of CoPA axons in the absence of midline crossing is dependent on PCP proteins

It has been previously established that CoPA axons do not need to cross the midline to become responsive to anterior–posterior guidance cues [15]. In *dcc* gene knockdown studies, many CoPA axons fail to cross the midline and remain ipsilateral, yet still extend axons anteriorly within the ipsilateral cord as if they had crossed the midline [15]. We therefore sought to determine whether the anterior guidance of uncrossed CoPA axons in *dcc* morphants is dependent on the PCP signaling pathway.

**Fig. 4:**
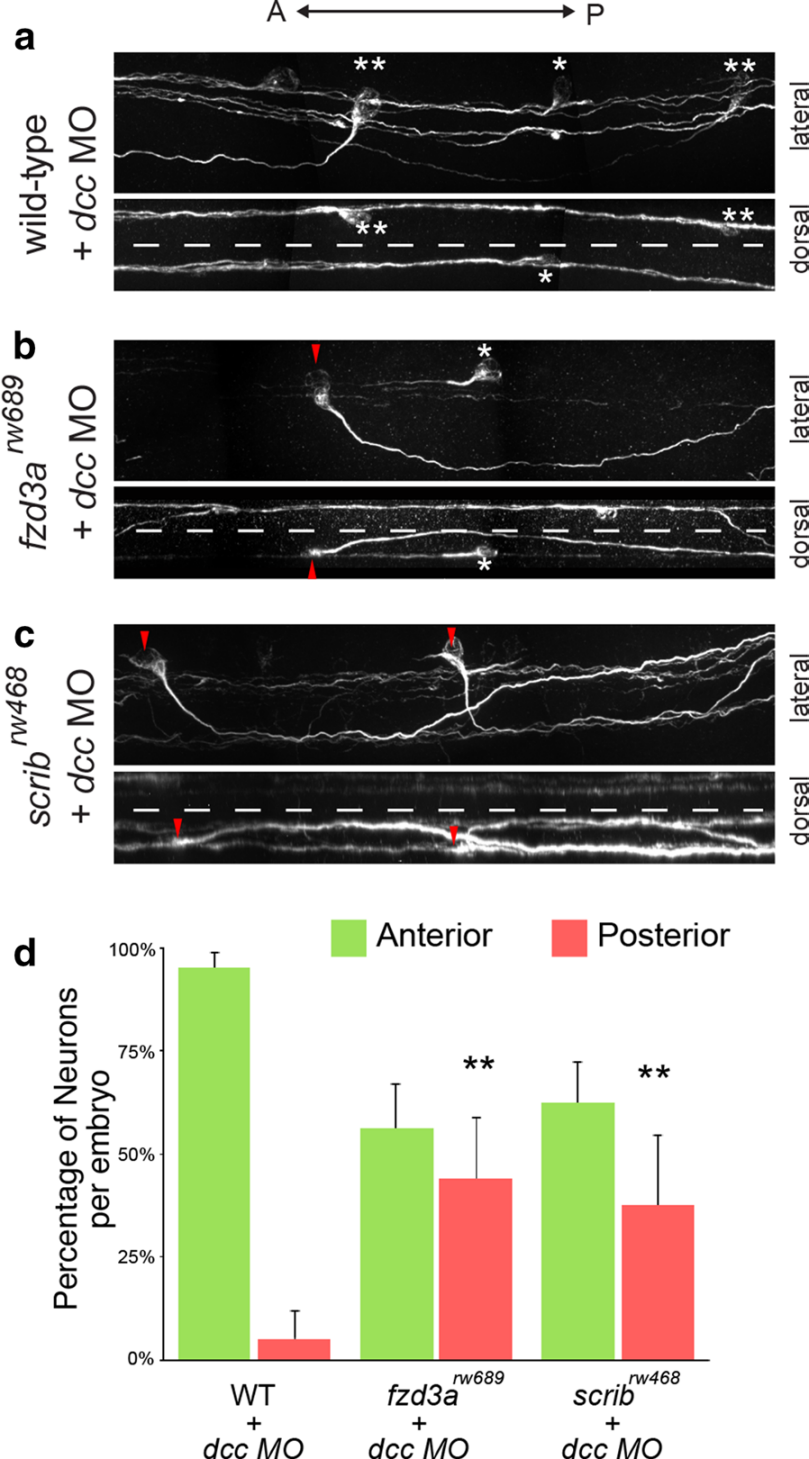
Anterior guidance of CoPA axons in the absence of midline crossing is dependent on PCP proteins. **a** CoPA axons fail to cross the midline in *dcc* knockdown embryos yet still undergo anterior growth. In the lateral view, *asterisk* indicates affected CoPA that pathfinds anteriorly with no ventral growth. *Double asterisk* indicates affected CoPA that displays weak ventral extension followed by projection dorsally and anteriorly without crossing the midline. Dorsal view of the same spinal cord demonstrates that affected CoPAs are prevented from crossing the midline (*dotted line*), **b**, **c** CoPA axons in *fzd3a* ^*rw689*^ and *scrib* ^*rw468*^ embryos injected with *dcc* morpholinos also fail to cross the midline. *Red arrowhead* indicates uncrossed CoPA axons that grow posteriorly inappropriately in *dcc*-deficient *fzd3a* ^*rw689*^ and *scrib* ^*rw468*^ embryos. *Asterisk* indicates uncrossed CoPA axon that projects anteriorly. Dorsal views of the same spinal cords demonstrate that posteriorly projecting CoPAs fail to cross the midline (*dotted line*), **d** quantification of the anterior or posterior trajectory of uncrossed CoPA neurons in *dcc*-deficient WT, *fzd3a* ^*rw689*^, and *scrib* ^*rw468*^ embryos. Error indicates SD. **p < 0.01 versus WT + *dcc* MO

We first confirmed that CoPA axons are capable of anterior axon pathfinding in the absence of midline crossing. As previously reported, two CoPA phenotypes were observed in *dcc* MO-injected embryos: (1) CoPA axons either completely fail to extend in the ventral direction or (2) they initially extend ventrally but fail to cross the midline (Fig. 4a). In both cases, the vast majority of uncrossed CoPA axons (95 ± 4%; 47 embryos; *n* = 138 neurons) project anteriorly within the ipsilateral spinal cord (Fig. 4a, d). To test whether the anterior guidance of ipsilateral CoPA axons in *dcc* knockdown embryos was dependent on PCP signaling, we injected *dcc* MOs into *fzd3a* ^*rw689*^ and *scrib* ^*rw468*^ mutants. We found that *dcc*-deficient uncrossed CoPA axons lose A–P directionality in the ipsilateral spinal cord of *fzd3a* ^*rw689*^ and *scrib* ^*rw468*^ mutants (Fig. 4b–d). In fact, approximately half of uncrossed CoPA axons turn posteriorly inappropriately in PCP mutants (*fzd3a* ^*rw689*^: 44 ± 15%, 18 embryos, *n* = 109 cells; *scrib* ^*rw468*^: 38 ± 17%, 29 embryos; *n* = 120 cells) (Fig. 4d). Taken together, these data support three major conclusions: (1) pre-crossing CoPA axons are responsive to anterior–posterior guidance cues, (2) anterior guidance is independent of midline crossing, and (3) anterior guidance, even in the absence of midline crossing, is dependent on components of the PCP signaling pathway.

## Discussion

Here, we establish the CoPA neuron in the zebrafish spinal cord as a model system to study the role of PCP in commissural axon guidance. The experiments described here extend earlier studies of PCP genes in the anterior guidance of commissural axons. In particular, we show that Frizzled3a and Vangl2 have evolutionarily conserved roles in spinal commissural growth cone steering along the anterior–posterior axis. Using loss-of-function PCP mutants, we have shown that CoPA axons are properly responsive to D–V guidance cues and navigate the midline appropriately, but are misguided along the anterior–posterior axis. With single neuron resolution, we show that loss of *fzd3a* or *vangl2* leads to randomized growth of CoPA axons along the A–P axis. We have also established a role for Scribble, an additional PCP protein, as essential for the proper anterior turning of commissural axons. We further show that even as pre-crossing CoPA axons extend ventrally to the floorplate they become responsive to A–P guidance cues and that the A–P bias of pre-crossing fibers is influenced by the function of PCP components. Even when midline crossing was prevented, we show that the anterior guidance exhibited by ipsilateral CoPA axons is dependent on PCP signaling components.

Accumulating evidence indicates that components of the PCP signaling pathway control the anterior guidance of spinal commissural axons. Loss of Fzd3, Vangl2, and Celsr3 in mice all lead to the randomized turning of post-crossing commissural axons along the anterior–posterior axis [25, 28]. Similar observations were made in dl1 commissural neurons in chick following siRNA knockdown of these PCP components [27, 29]. Our findings that CoPA axons in the *fzd3a and vangl2* mutant zebrafish spinal cord randomly turn anterior or posterior support an evolutionarily conserved role for Fzd3a and Vangl2 in regulating A–P guidance of commissural axons among vertebrates. We extend these findings to include Scrib as essential for the proper anterior turning of spinal commissural axons. Loss of function studies in both Drosophila and vertebrates have described Scrib as a determinant of planar cell polarity [18, 47]. In mammals, loss of Scrib leads to defects in neural tube closure that arise due to defective convergence extension cell movement and a disrupted orientation of stereociliary bundles of inner ear hair cells [18, 48]. Furthermore, Scrib genetically and physically interacts with Vangl2 during these processes [18, 19, 48]. In the nervous system, Scrib, similar to other core PCP proteins, is required for the asymmetric posterior localization of motile cilia on neuroepithelial cells and the migration of facial branchiomotor neurons in the developing hindbrain [19, 23, 24].

What is the role of Scribble in PCP-mediated growth cone steering? Scribble has been found to be part of a Vangl2 complex, where it physically interacts with the C-terminus of Vangl2 [47–49]. Recently, EGFP-Vangl2 was found to be enriched at the tips of growth cone filopodia [28, 50]. Specifically, growth cone filopodia that are elongating or stable have high levels of Vangl2 at their tips [28]. One possibility is that Vangl2-Scrib interactions regulate actin dynamics in growth cones. In both migrating cells and neuronal dendritic spines, Scrib recruits the G-protein coupled receptor interacting protein 1 (GIT1)/*β*-p21-activated kinase- (PAK-) interacting exchange factor (*β*-PIX)/PAK complex to the plasma membrane to regulate actin dynamics through its influence on Rac activity [51–57]. An alternative hypothesis is that a Vangl2-Scrib interaction facilitates Fzd3 endocytosis and recycling in commissural growth cones. Frizzled receptor endocytosis has been shown to be important for PCP signaling [58–60]. In commissural neurons, Fzd3 undergoes endocytosis at filopodial tips in response to Wnt5a [50]. When Vangl2 is present, Wnt5a-mediated PCP signal transduction is increased and the amount of surface level of Fzd3 is decreased, presumably because Vangl2 promotes Fzd3 internalization [28]. Interestingly, Scrib has been implicated in trafficking and recycling of endocytic cargo in a variety of contexts [51, 61, 62] and could regulate Wnt-mediated Fzd3 internalization and recycling in growth cones.

What activates the PCP pathway to control anterior turning of commissural axons? The role of Wnt ligands as axon guidance cues have been reported for a number of neuronal populations in the mammalian CNS [63]. Exogenously supplied Wnts are sufficient to attract commissural axons, including Wnt4, that in mice is expressed in an anterior–high, posterior-low gradient in the spinal cord at the time that commissural axons are actively pathfinding [25]. In chick, RNAi-mediated knockdown of two Wnt ligands, Wnt5a and Wnt7a, lead to a defect in the rostral turning of commissural axons, however, these Wnt ligands are not expressed in a gradient [26]. Rather, SHH expressed in a descending gradient from posterior to anterior controls the graded expression of the Wnt antagonist SFRP1 in the chick spinal cord that creates an opposing Wnt-activity gradient [26]. Nevertheless, in both mouse and chick, loss of Fzd3 results in randomized A–P pathfinding in which axons turn anterior or posterior, with a significant proportion of axons appearing to stall, consistent with the model that Wnt-Fzd3 signaling promotes anterior growth cone steering after midline crossing [25, 27]. With single neuron resolution, we show that loss of *fzd3a* or *vangl2* also leads to randomized growth along the A–P axis, yet CoPA axons do not stall or wander. Rather, they grow for long distances in either the anterior (correct) or posterior (incorrect) direction. It is clear that PCP signaling components are required for the interpretation of anterior–posterior guidance cues by CoPA axons. However, in this system, the absence of stalling or wandering of CoPA axons in *fzd3a* mutants may be at odds with the model that Wnt ligands expressed in a gradient simply attract CoPA axons anteriorly. Our findings support the presence of additional guidance molecules that regulate A–P guidance of CoPA axons, and suggest the possibility that PCP components are required for the proper interpretation of other extrinsic guidance cues.

For many neural circuits, growth navigation relies on timely changes in responsiveness from one guidance cue to another for proper navigation past intermediate targets [3, 64, 65]. For instance, mammalian commissural neurons become responsive and sensitized to floorplate-derived repellants Slits and Semaphorins, only after crossing the midline [11]. Likewise, observations in mouse and chick indicate that responsiveness to Wnt-Fzd signaling for anterior turning increased only after midline crossing [25, 26]. In mice, no defects in the guidance of pre-crossing tracts of dorsal commissural axons were found in Fzd3 or Vangl2 knockout mice [25, 28]. Moreover, COS cells engineered to secrete Wnt4 were sufficient to attract commissural axons, but only after midline crossing in spinal cord explants [25]. A switch in responsiveness to Wnt-PCP signaling may be controlled by PI3 kinase activity, since blocking PI3 kinase signaling caused A–P randomization of commissural axons whereas overexpression of the catalytic subunit p110γ switched on Wnt-mediated attractiveness in advance of crossing the midline [66]. In contrast, responsiveness of CoPA axons to A–P guidance cues is not dependent on midline-crossing [15]. CoPA axons pathfind anteriorly even when midline crossing is prevented by *dcc* knockdown [15]. Our results show that the initial anterior orientation of axon outgrowth of CoPA axons is influenced by PCP signaling components, since a significant number of CoPA axons grow ventral-posteriorly in PCP mutants. We further show that the anterior guidance of uncrossed CoPA axons in *dcc* morphants is dependent on PCP signaling. Thus, in the absence of both PCP signaling and midline crossing ipsilateral CoPA axons make randomized decisions to grow anteriorly or posteriorly within the ipsilateral cord. These observations are consistent with the idea that CoPA axons are capable of responding to A–P guidance cues and that they require PCP components to do so.

## Conclusion

In summary, these experiments establish CoPA axons as a model system to investigate the mechanism of PCP signaling in commissural axon guidance. Our results support three major conclusions, (1) PCP signaling components are not required for axon growth per se, (2) loss of PCP components does not affect dorsal–ventral guidance decisions or midline crossing by commissural axons, (3) PCP signaling components are essential for anterior–posterior guidance of CoPA axons both prior to and after midline crossing.

## Abbreviations

CoPA: commissural primary ascending
DLF: dorsal longitudinal fasciculus
A–P: anterior–posterior
Hpf: hours post fertilization
DCC: deleted in colorectal cancer
MO: morpholino oligonucleotide
fzd3a: Frizzled3a
vangl2: van-gogh like 2
scrib: Scribble
pk: prickle
